# Toxic Epidermal Necrolysis and Mortality: A Danish Cohort Study With 30 Years of Follow‐Up

**DOI:** 10.1111/1346-8138.70040

**Published:** 2025-10-27

**Authors:** Ida M. Heerfordt, Magnus Middelboe, Ann Hærskjold, Anna Horwitz, Rasmus Huan Olsen, Henrik Horwitz

**Affiliations:** ^1^ Department of Clinical Pharmacology Copenhagen University Hospital—Bispebjerg and Frederiksberg Copenhagen Denmark; ^2^ Department of Geriatric and Palliative Medicine Copenhagen University Hospital—Bispebjerg and Frederiksberg Copenhagen Denmark; ^3^ Department of Clinical Medicine University of Copenhagen Copenhagen Denmark; ^4^ Department of Dermatology Copenhagen University Hospital—Bispebjerg and Frederiksberg Copenhagen Denmark

**Keywords:** adverse drug reactions, dermatology, mortality, Stevens–Johnson syndrome, survival analysis, toxic epidermal necrolysis

## Abstract

Toxic epidermal necrolysis (TEN) is a rare, life‐threatening dermatologic condition, typically triggered by medications. While increased short‐term mortality in this group is well documented, few studies have quantified this risk over extended follow‐up. We aimed to examine both short‐ and long‐term mortality and assess whether excess mortality persists beyond the acute phase. We conducted a nationwide, register‐based matched cohort study using Danish health registries. Patients with a first‐time hospital diagnosis of TEN between 1995 and 2024 were included and matched 1:100 on age and sex with population controls. Short‐term mortality (30‐ and 90‐day) was calculated, and long‐term mortality was assessed overall and in a landmark analysis restricted to 2‐year survivors with newly matched controls. We identified 145 individuals hospitalized with TEN and matched them to 14 500 population controls. The mean age was 56 years, and half were female. Short‐term mortality was high, with 28% dying within 90 days. Median survival was 4.77 years (95% CI: 2.02–9.46) for TEN patients versus 25.96 years (95% CI: 25.19–27.20) for controls. Two years after diagnosis, TEN patients still had almost triple the mortality risk compared to controls (HR 3.30; 95% CI: 2.34–4.66). Excess mortality was also observed among patients without any recorded comorbidities at the time of diagnosis, indicating that the increased risk was not solely attributable to preexisting health conditions. Most deaths were due to natural causes; fewer than five were attributed to unnatural or unknown causes. In conclusion, this study quantifies the long‐term reduction in survival following TEN. Our estimates captured the real‐world excess mortality burden of TEN, arising from the acute disease, sequelae, or comorbidities, thus providing a clinically relevant picture of long‐term prognosis. The increased mortality risk was not explained by baseline comorbidity alone, underscoring that TEN itself contributes to a poorer long‐term prognosis.

## 1 Introduction

Toxic epidermal necrolysis (TEN) is a rare but serious skin reaction, usually triggered by drugs [1, 2]. It causes extensive skin cell death and detachment and there is currently no cure [1, 3]. First‐line management involves the immediate discontinuation of all nonessential medications, followed by supportive care [1, 3, 4]. In some cases, immunosuppressive treatments such as ciclosporin or TNF‐α inhibitors may be considered [1, 3]. However, understanding the immune mechanisms behind TEN has identified potential targets for future treatments [1, 3].

TEN is known to have a high short‐term mortality rate, with up to 35% dying within the first year [5, 6, 7, 8]. Although TEN is regarded as an acute condition, studies suggest that many survivors continue to face serious health problems and a higher risk of dying in the years after discharge [8, 9, 10]. These include damage to mucous membranes, eyes, mental health, and other organ systems [1, 8, 11].

As treatment options for the acute phase improve and more patients survive, it becomes more important to understand long‐term sequelae and prognosis [12]. However, little is known about long‐term survival and causes of death in this group [7, 8, 11, 13].

This study aimed to examine long‐term mortality and causes of death in a nationwide cohort of patients hospitalized with TEN. To address whether excess mortality could be explained by underlying health status, we also performed a subanalysis restricted to patients without any recorded comorbidity at baseline. Our estimates capture the real‐world excess mortality burden associated with TEN, whether driven by the acute disease itself, its sequelae, or underlying comorbidities, thereby providing a clinically relevant picture of long‐term prognosis.

## 2 Methods

This was a nationwide register‐based matched cohort study using Danish health data collected over a 30‐year period, from January 1, 1995, to April 29, 2025.

The study was approved by the Danish Data Protection Agency (p‐2024‐15 764) and the Danish National Board of Health (FSEID‐00007077). As the study was registry‐based and involved anonymized data, informed consent was not required under Danish law. Due to Danish data protection regulations, results based on fewer than five individuals could not be reported.

### 2.1 Data Sources

We used data from multiple national Danish health registries, which were linked at the individual level using the unique personal identification number assigned to all Danish residents. The following registries were used:

– The Danish Civil Registration System [14]: Contains data on date of birth, sex, vital status, date of death, and emigration status for all residents of Denmark.
– The Danish Register of Causes of Death [15]: Records all registered causes of death in Denmark, based on death certificates. Each death is classified using ICD‐10 codes, and causes are categorized as natural (A–R) or unnatural (S–Y).
– The Danish National Patient Register [16]: Contains detailed records of all hospital contacts in Denmark, including inpatient admissions, outpatient visits, and emergency room visits. It includes diagnostic codes using International Classification of Diseases, 10th Revision (ICD‐10) for each contact, enabling tracking of diseases and comorbidities over time. The ICD‐10 codes for TEN can only be assigned by hospital physicians, most often in departments of dermatology, internal medicine, acute admission units, intensive care, or burn units. Diagnoses are typically made by experienced specialists based on clinical presentation, and while histopathological confirmation is not mandatory, it is frequently performed to support the diagnosis. A previous validation study has shown a high diagnostic accuracy of TEN coding in the Danish National Patient Register under these conditions [17].

### 2.2 Study Population

In Denmark patients with suspected TEN are admitted free of charge to public hospitals, which are accessible to all Danish residents. We identified all individuals with a first‐time inpatient diagnosis of TEN (ICD‐10: L51.2) recorded in the Danish National Patient Register from January 1, 1995, to December 31, 2024. Only the first registered episode was included to avoid counting readmissions. The index date was defined as the date of hospital admission for TEN. Each TEN case was matched 1:100 with population controls from the Danish Civil Registration System based on year of birth, sex, and index date. Controls were required to be alive and residing in Denmark on the index date and to have no previous diagnosis of TEN. Matched controls were assigned the same index date as their corresponding case.

To assess long‐term mortality independent of early deaths, we further identified all TEN patients who survived at least 2 years after the index date. For each surviving TEN patient, 50 new controls were randomly resampled from the original pool of population controls, restricted to those who were alive at the 2‐year point. These resampled controls were matched on year of birth and sex.

### 2.3 Comorbidity

Comorbidity burden was evaluated for each study participant based on all primary and secondary hospital diagnoses recorded in the Danish National Patient Register in the 12 months prior to the index date. These diagnoses were used to calculate the Charlson Comorbidity Index (CCI) [18] based on a standard algorithm adapted to Danish ICD‐10 codes [19]. The CCI includes diagnoses such as myocardial infarction, congestive heart failure, peripheral vascular disease, cerebrovascular disease, dementia, chronic pulmonary disease, rheumatologic disease, peptic ulcer disease, liver disease, diabetes, hemiplegia or paraplegia, renal disease, malignancy, leukemia, lymphoma, and AIDS. CCI scores were categorized as 0, 1–2, or ≥ 3 points.

### 2.4 Outcomes

The primary outcome was all‐cause mortality, defined as death from any cause between the index date and the end of follow‐up (April 29, 2025), or emigration, whichever occurred first. Vital status and date of death were obtained from the Danish Civil Registration System.

Secondary outcomes included:

– Short‐term all‐cause mortality, measured as death occurring within 30 and 90 days of the index date.
– As a supplementary analysis, we examined outcomes among individuals with no recorded comorbidities (CCI = 0) at index.
– Long‐term all‐cause mortality among patients who survived at least 2 years after the index date, to assess mortality independent of early, acute‐phase deaths. Follow‐up for this restricted cohort began 2 years after the index date and continued until death, emigration, or until the end of the study period (April 29, 2025).
– Cause‐specific mortality among all deceased TEN patients, classified as natural or unnatural based on ICD‐10 codes from the Danish Register of Causes of Death, regardless of the time of death.

### 2.5 Statistical Analysis

All statistical analyses were conducted using SAS version 9.4 (SAS Institute Inc., Cary, NC, USA). Statistical significance was defined as a two‐sided *p*‐value < 0.05.

To assess short‐term mortality, the proportion of deaths within 30 and 90 days following the index date was calculated for both TEN patients and controls. Comparisons were performed using chi‐square tests, and predictors of 90‐day mortality within the TEN cohort were examined using bivariate comparisons. These included mean age and mean CCI scores (Wilcoxon rank‐sum tests), as well as categorical CCI distribution (chi‐square).

For long‐term survival, cumulative survival functions were estimated using Kaplan–Meier survival curves, with comparison between TEN cases and controls performed via the log‐rank test. Median survival times with 95% confidence intervals (CIs) were calculated for both groups.

Due to the high short‐term mortality, the proportional hazards assumption was violated. So, for the analysis of long‐term mortality beyond the early high‐risk period, we included TEN patients who were alive 2 years after the index date, along with newly resampled controls from the original control pool, matched on year of birth and sex and also alive at the 2‐year point. Follow‐up for this analysis began 2 years after the index date. As a sensitivity analysis, we restricted the cohort to individuals with no recorded comorbidities (CCI = 0) at index. Long‐term survival was analyzed using Kaplan–Meier curves and the log‐rank test was employed for significance testing. Hazard ratios for mortality were estimated using conditional Cox proportional hazards models. *E*‐values were calculated; see Supporting Information methods.

Cause‐specific mortality was assessed descriptively.

## 3 Results

### 3.1 Study Population and Index Characteristics

Between January 1, 1995, and December 31, 2024, we identified 145 individuals with a first‐time hospital diagnosis of TEN. The mean age at admission among TEN cases was 55.8 years (SD 24.1), and 50.3% were female. Patients were matched to 14 500 population controls. The distribution of index characteristics is presented in Table 1. Comorbidity burden was substantially higher among TEN patients compared to controls, with a mean CCI of 1.12 versus 0.12. More than 47% of TEN patients had a CCI ≥ 1 compared to only 7% of controls.

**TABLE 1:** Index characteristics and short‐term mortality of patients with TEN and matched controls.

Characteristic	TEN cases (*n* = 145)	Controls (*n* = 14 500)
Age at index, mean (SD)	55.80 (24.13)	55.81 (24.05)
Index year		
Mean (SD)	2008 (8)	2008 (8)
Minimum	1995	1995
Maximum	2024	2024
Sex, *n* (%)		
Female	73 (50.34%)	7300 (50.34%)
Male	72 (49.66%)	7200 (49.66%)
CCI at index, mean	1.12	0.12
CCI at index, *n* (%)		
0	76 (52.41%)	13 460 (92.83%)
1–2	51 (35.17%)	946 (6.52%)
3+	18 (12.41%)	94 (0.65%)
Mortality time point, *n* (%)		
30‐day mortality	28 (19.31%)	29 (0.20%)
90‐day mortality	41 (28.28%)	99 (0.68%)

Abbreviations: CCI, Charlson Comorbidity Index; TEN, toxic epidermal necrolysis.

### 3.2 Short‐Term Mortality

Short‐term mortality among TEN cases was high, with 19.3% dying within 30 days and 28.3% within 90 days of diagnosis. In contrast, only 0.2% and 0.7% of controls died within the same time frames, respectively (Table 1). The main predictors of 90‐day mortality included older age and higher comorbidity burden. TEN patients who died within 90 days had a mean age of 70.3 years, compared to 50.1 years among TEN survivors (*p* < 0.0001). Higher CCI was also significantly associated with early mortality (mean 1.56 vs. 0.95, *p* = 0.01; Table 2).

**TABLE 2:** Predictors of 90‐day mortality among patients with TEN.

Predictor	TEN survivors (*n* = 104)	TEN deceased (*n* = 41)	*p*
Age, mean (SD)	50.07 (24.20)	70.34 (16.92)	< 0.0001
Year of TEN diagnosis, mean (SD)	2007.69 (8.43)	2008.68 (7.51)	0.49
Female sex, *n* (%)	55 (52.88%)	18 (43.90%)	0.33
CCI at index, mean (SD)	0.95 (1.60)	1.56 (1.86)	0.0104
CCI = 0, *n* (%)	62 (59.62%)	14 (34.15%)	0.0131
CCI = 1–2, *n* (%)	31 (29.81%)	20 (48.78%)	
CCI ≥ 3, *n* (%)	11 (10.58%)	7 (17.07%)	

Abbreviations: CCI, Charlson Comorbidity Index; TEN, toxic epidermal necrolysis.

### 3.3 Long‐Term Survival

**FIGURE 1:**
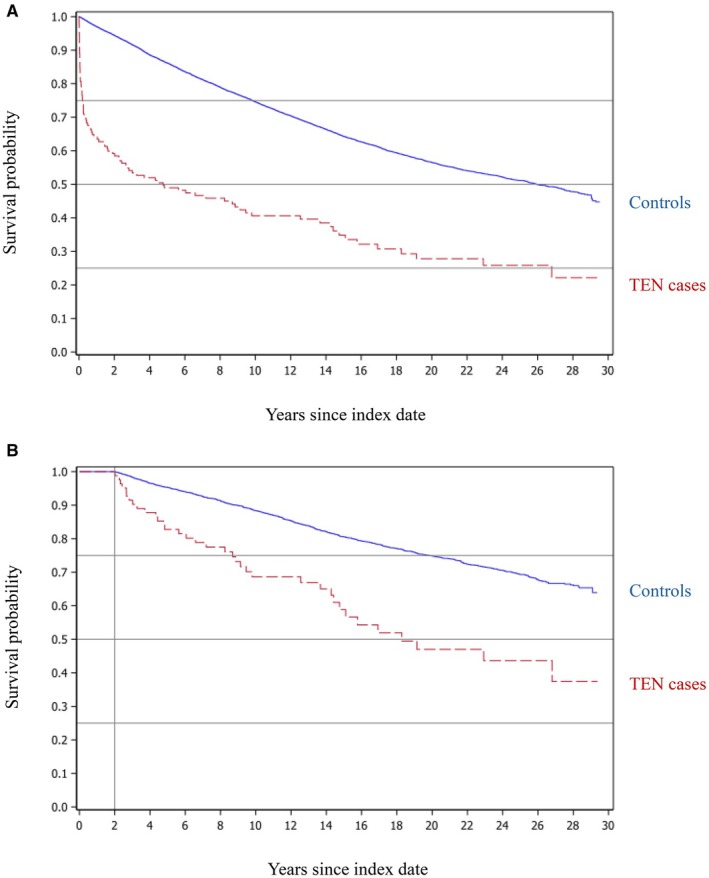
Kaplan–Meier survival curves for patients with a history of toxic epidermal necrolysis (TEN) and matched population controls. (A) Overall survival from the index date (hospital admission for TEN cases and the corresponding matched date for controls). Follow‐up began at the index date. (B) Survival among TEN patients and matched controls who were alive 2 years after the index date. For this landmark analysis, new controls were resampled and re‐matched on age and sex. Follow‐up began 2 years after the index date.

Long‐term survival is presented in Figure 1A. The median survival time was 4.77 years (95% CI: 2.02–9.46) among patients with TEN, compared to 25.96 years (95% CI: 25.19–27.20) among controls (log‐rank *p* < 0.001). Due to the high short‐term mortality, the proportional hazards assumption is violated. Since mortality is extensive among elderly TEN patients, a significant age difference between TEN patients and controls develops over time. At index, the age difference between TEN patients and controls is zero, but after 2 years of follow‐up, the surviving TEN patients are, on average, 8 years younger than the surviving controls (8.19 years; 95% CI: 2.79–13.60). This age difference further complicates the assessment of the Kaplan–Meier curve and may lead to the erroneous impression that after the acute phase the long‐term mortality among TEN patients resembles that of the controls.

Thus, to get a better assessment of long‐term mortality beyond the early high‐risk period, we restricted the analysis to patients and matched controls who were alive 2 years after the index date. Index characteristics for this subgroup are presented in Table S1. Kaplan–Meier survival curves for this restricted cohort are shown in Figure 1B. In this analysis, overall mortality remained substantially higher among patients with TEN compared to controls, with a crude HR of 3.30 (95% CI: 2.34–4.66).

When we restricted our analysis to individuals with a CCI of 0 at index, TEN patients still had an excess risk of dying (HR 2.82; 95% CI: 1.75–4.57). Descriptive characteristics for this subgroup are presented in Tables S2 and S3. Figures S1 and S2 show Kaplan–Meier survival curves and *E*‐values for these individuals with CCI of 0 at index.

### 3.4 Causes of Death

Among deceased TEN patients, the vast majority died from natural causes. Fewer than five deaths were attributed to unknown or unnatural causes such as accidents, suicide, or violence. Thus, no further investigation was conducted within this area.

## 4 Discussion

In this nationwide cohort study spanning three decades, we found that individuals who had been hospitalized with TEN experienced significantly increased long‐term mortality compared to age‐ and sex‐matched population controls. While elevated short‐term mortality is well known in TEN [5, 6, 7, 8], our study adds to the limited evidence on long‐term mortality by quantifying the excess risk over a 30‐year follow‐up period [5, 7, 8], demonstrating a median overall survival nearly 20 years shorter than expected.

To better assess long‐term mortality beyond the high‐risk early period, we restricted the analysis to patients and controls who were alive 2 years after the index date. Due to the substantial early mortality among TEN patients, the original matching could not be maintained. Therefore, new controls were resampled from the original pool to ensure comparability on age, sex, and vital status at the 2‐year point.

In this restricted analysis, patients with TEN continued to have substantially higher mortality compared to controls. These findings indicate that TEN may have lasting effects on health beyond the acute phase, especially since patients without chronic comorbidities at baseline also displayed markedly elevated long‐term mortality.

A strength of our study is the use of nationwide, validated registries with virtually complete follow‐up, enabling precise survival analyses and cause‐of‐death classification. The diagnostic coding of TEN in Danish health registries has previously been validated with high accuracy [17]. Although Stevens–Johnson syndrome (SJS) and TEN are part of the same clinical spectrum and share pathophysiological features [1], we deliberately restricted our cohort to patients with a diagnosis of TEN due to concerns about diagnostic validity. A previous Danish study demonstrated that while the registration of TEN in the Danish National Patient Register is highly specific and reliably validated through clinical and histological criteria, the accuracy for SJS is substantially lower, with many cases representing other dermatological conditions [17].

Our study has limitations that warrant consideration. The small number of TEN cases, despite the nationwide scope and long follow‐up period, reflects the rarity of the condition and limits the statistical power, particularly for subgroup analyses. It also restricts the ability to report detailed results, as data protection regulations prevent disclosure of very small numbers to protect patient anonymity. Furthermore, only patients with a hospital‐recorded diagnosis of TEN were included, which means individuals who died before reaching hospital care were not captured. Although likely rare, their exclusion may lead to a slight underestimation of overall mortality. Finally, our study describes the real‐world prognosis of patients with TEN, but it does not allow us to determine with certainty which specific mechanisms or conditions were responsible for the observed excess mortality. The findings indicate that comorbidities likely contribute to the mortality burden, yet the excess risk was also evident among patients without any recorded comorbidities, suggesting that TEN itself plays an important role in the long‐term prognosis.

While the CCI captures most major chronic diseases, including dementia, other mental health conditions, chronic pain, and obesity are not explicitly represented and may contribute to overall vulnerability and mortality. However, the primary objective of this study was prognostic rather than causal. Our analyses were designed to describe the overall mortality burden among patients with TEN, encompassing both the acute effects of the disease and the broader health context in which it occurs. Accordingly, chronic and malignant conditions were considered integral components of the patients' health status rather than confounders to be adjusted away. Ultimately, the enduring excess mortality after TEN serves as a reminder that survival from the acute event does not equal recovery.

## Ethics Statement

The study was registry‐based and conducted using anonymized data, in accordance with Danish law. The study was approved by the Danish Data Protection Agency (ref. no. P‐2024‐15 764) and the Danish National Board of Health (ref. no. FSEID‐00007077).

## Consent

The authors have nothing to report.

## Conflicts of Interest

The authors declare no conflicts of interest.

## Supporting information


**Data S1:** jde70040‐sup‐0001‐DataS1.docx.

## Data Availability

The data that support the findings of this study are available on request from the corresponding author. The data are not publicly available due to privacy or ethical restrictions.
