# Supplementary material for: Toxic Epidermal Necrolysis and Mortality: A Danish Cohort Study With 30 Years of Follow‐Up
Source: J Dermatol. 2025 Oct 27;53(1):35–40. doi: 10.1111/1346-8138.70040 (PMC12784798; doi:10.1111/1346-8138.70040)

**Supplementary material**

**Toxic Epidermal Necrolysis and Mortality: A Danish Cohort Study with 30 Years of Follow-Up**

Ida M. Heerfordt^1,2^, Magnus Middelboe^1^, Ann Hærskjold^3,4^, Anna Horwitz^3^, Rasmus Huan Olsen^1,3^, Henrik Horwitz^1,3^

^1^ Department of Clinical Pharmacology, Copenhagen University Hospital - Bispebjerg and Frederiksberg, Denmark

^2^ Department of Geriatric and Palliative Medicine, Copenhagen University Hospital - Bispebjerg and Frederiksberg, Denmark

^3^ Department of Clinical Medicine, University of Copenhagen, Denmark

^4^ Department of Dermatology, Copenhagen University Hospital - Bispebjerg and Frederiksberg, Denmark

**Supplementary methods**

**E-value calculation**

The E-value was calculated as:

E-value = HR + square root of [HR × (HR − 1)]

where HR denotes the hazard ratio.

**Supplementary results**

**Table S1.** Index characteristics of TEN patients and matched controls among two-year survivors (all CCI strata). This table presents descriptive characteristics of TEN patients (n = 83) and matched controls (n = 4,150) who were alive two years after the index date. Patients and controls were matched on sex and year of birth 1:50. Due to data protection restrictions, comorbidity was summarized as CCI = 0 vs. CCI ≥ 1. Abbreviations: CCI = Charlson Comorbidity Index, TEN = toxic epidermal necrolysis.

| **Characteristic** | **TEN cases (n = 83)** | **Controls (n = 4150)** |
| --- | --- | --- |
| **Age at index, mean (SD)** | 46.1 (24.7) | 46.1 (24.6) |
| **Index year** |  |  |
| Mean (SD) | 2007 (8) | 2007 (8) |
| Minimum | 1995 | 1995 |
| Maximum | 2023 | 2023 |
| **Sex, %** |  |  |
| Female | 55.42% | 55.42% |
| Male | 44.58% | 44.58% |
| **CCI at index, mean** | 0.54 | 0.07 |
| **CCI at index, n (%)** |  |  |
| 0 | 55 (66.27%) | 3,969 (95.64%) |
| 1+ | 28 (33.73%) | 181 (4.36%) |

**Table S2.** This table displays characteristics of TEN patients and their matched controls who had no recorded comorbidities (CCI = 0) at index. The cohort includes all patients and controls regardless of subsequent survival. Abbreviations: CCI = Charlson Comorbidity Index, TEN = toxic epidermal necrolysis.

| **Characteristic** | **TEN cases (n = 76)** | **Controls (n = 3,800)** |
| --- | --- | --- |
| **Age at index, mean (SD)** | 48.9 (27.2) | 48.9 (27.2) |
| **Index year** |  |  |
| Mean (SD) | 2006 (8) | 2006 (8) |
| Minimum | 1996 | 1996 |
| Maximum | 2024 | 2024 |
| **Sex, %** |  |  |
| Female | 52.63% | 52.63% |
| Male | 47.37% | 47.37% |
| **CCI at index** | 0 | 0 |

**Table S3.** This table summarizes the subgroup of TEN patients and matched controls with no index comorbidity (CCI = 0), who were alive two years after the index date. Abbreviations: CCI = Charlson Comorbidity Index, TEN = toxic epidermal necrolysis.

| **Characteristic** | **TEN cases (n = 55)** | **Controls (n = 2,750)** |
| --- | --- | --- |
| **Age at index, mean (SD)** | 40.2 (24.9) | 40.2 (24.8) |
| **Index year** |  |  |
| Mean (SD) | 2006 (8) | 2006 (8) |
| Minimum | 1996 | 1996 |
| Maximum | 2023 | 2023 |
| **Sex, %** |  |  |
| Female | 52.73% | 52.73% |
| Male | 47.27% | 47.27% |
| **CCI at index** | 0 | 0 |

**Figure S1.** Kaplan–Meier survival curves for patients with TEN and matched controls, restricted to individuals with no baseline comorbidity (CCI = 0). The red dashed line represents TEN patients. The blue solid line represents controls. The hazard ratio (TEN vs. controls) for all-cause mortality during the first two years was 9.04 (95% CI 5.66–14.47), corresponding to an E-value of 17.56 (95% CI 10.79–28.43). Abbreviations: CCI = Charlson Comorbidity Index, TEN = toxic epidermal necrolysis.


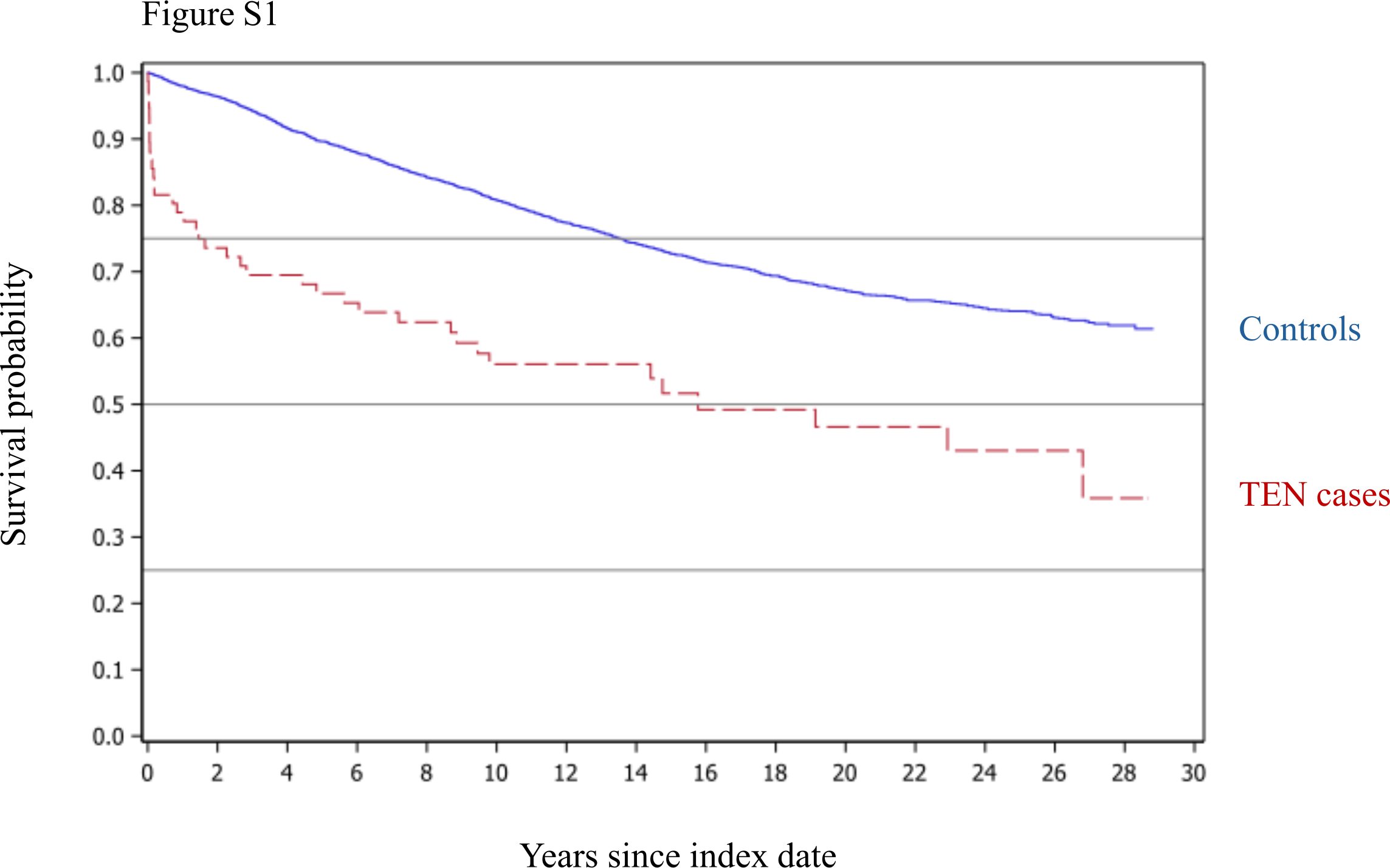


**Figure S2.** Kaplan–Meier survival curves for TEN patients and matched controls with CCI = 0 at index, restricted to individuals who survived at least two years after the index date. The red dashed line represents TEN patients. The blue solid line represents controls. The hazard ratio (TEN vs. controls) for subsequent all-cause mortality (2–30 years of follow-up) was 2.82 (95% CI 1.75–4.57), corresponding to an E-value of 5.09 (95% CI 2.90–8.61). Abbreviations: CCI = Charlson Comorbidity Index, TEN = toxic epidermal necrolysis.


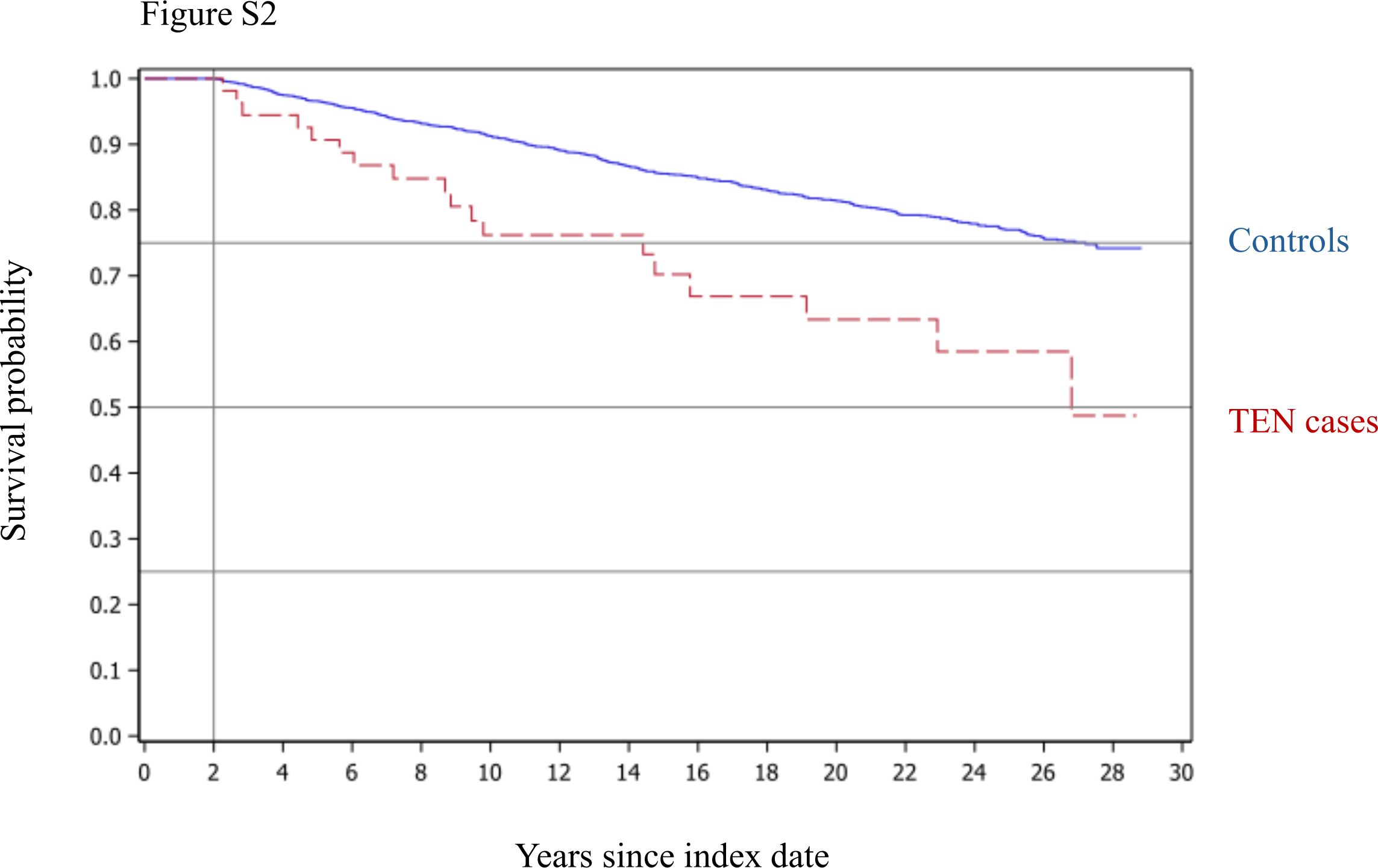

Supplement: Supplementary file 1 — Data S1: jde70040‐sup‐0001‐DataS1.docx. [file JDE-53-35-s001.docx]
